# Communication Pattern Changes Along With Declined IGF1 of Immune Cells in COVID-19 Patients During Disease Progression

**DOI:** 10.3389/fimmu.2021.729990

**Published:** 2022-01-14

**Authors:** Min Zhao, Zhen Liu, Fei Shao, Wenjing Zhou, Zhu Chen, Pengyan Xia, Shuo Wang, Penghui Yang

**Affiliations:** ^1^ Chinese Academy of Sciences (CAS) Key Laboratory of Pathogenic Microbiology and Immunology, Institute of Microbiology, Chinese Academy of Sciences, Beijing, China; ^2^ University of Chinese Academy of Sciences, Beijing, China; ^3^ National Clinical Research Center for Infectious Diseases, Fifth Medical Center of Chinese People’s Liberation Army (PLA) General Hospital, Beijing, China; ^4^ Department of Immunology, School of Basic Medical Sciences, Peking University, Beijing, China; ^5^ National Health Commission (NHC) Key Laboratory of Medical Immunology, Peking University, Beijing, China; ^6^ Key Laboratory of Molecular Immunology, Chinese Academy of Medical Sciences, Beijing, China

**Keywords:** communication pattern, IGF1, immune cells, single-cell sequencing, COVID-19

## Abstract

The emergence of severe acute respiratory syndrome coronavirus 2 (SARS-CoV-2), which causes the coronavirus disease 2019 (COVID-19) pandemic, represents a global crisis. Most patients developed mild/moderate symptoms, and the status of immune system varied in acute and regulatory stages. The crosstalk between immune cells and the dynamic changes of immune cell contact is rarely described. Here, we analyzed the features of immune response of paired peripheral blood mononuclear cell (PBMC) samples from the same patients during acute and regulatory stages. Consistent with previous reports, both myeloid and T cells turned less inflammatory and less activated at recovery phase. Additionally, the communication patterns of myeloid-T cell and T-B cell are obviously changed. The crosstalk analysis reveals that typical inflammatory cytokines and several chemokines are tightly correlated with the recovery of COVID-19. Intriguingly, the signal transduction of metabolic factor insulin-like growth factor 1 (IGF1) is altered at recovery phase. Furthermore, we confirmed that the serum levels of IGF1 and several inflammatory cytokines are apparently dampened after the negative conversion of SARS-CoV-2 RNA. Thus, these results reveal several potential detection and therapeutic targets that might be used for COVID-19 recovery.

## Introduction

Since severe acute respiratory syndrome coronavirus 2 (SARS-CoV-2) broke out at the end of 2019, it has caused more than one hundred million coronavirus disease 2019 (COVID-19) infections with over three million deaths (1). Typical symptoms of COVID-19 include fever and dry cough while uncommon symptoms such as muscle pain, headache, diarrhea also appear in patients (2, 3). Ground-glass opacity almost presented in all CT images of patients (4). Approximately 20% of the patients developed severe diseases. Understanding the immunological features and the signal transduction of the immune system of COVID-19 patients is crucial for the development of therapeutic strategies.

For the infection of SARS-CoV-2, the host undergoes acute and regulatory stage along with various immune features. Type I interferon response emerges rapidly resulting in the upregulation of interferon stimulated genes (ISGs) in coronavirus infection (5). As previously reported, ISGs were upregulated in monocytes accompanied with a decreased level of HLA-DR (6–8). In CD14^+^ inflammatory monocytes, inflammatory related genes, chemokines, and ISGs were highly expressed, while anti-inflammatory genes were downregulated (9). Dysfunctional myeloid cells might be correlated with acute respiratory distress syndrome (ARDS), cytokine release syndrome (CRS), and lymphopenia, which is commonly observed in COVID-19 patients with severe symptoms (10). Besides, T and NK cells exhibited high expression of ISGs (6, 9, 11).

Lymphocytes and monocytes were observed infiltrating in lungs in fetal cases (12). Similarly, in ACE2-transgenic mice, macrophages and lymphocytes could be observed in the pulmonary interstitium (13), indicating a crosstalk of lymphocyte and myeloid cell in pathogenesis. Neutrophil to lymphocyte ratio was considered as a potential prognostic marker of disease severity (14, 15). SARS-CoV-2-specific T cells played an important role in antiviral response and were affected by monocytes (16). However, it is still unknown how monocytes and lymphocyte subsets interact to influence clinical outcome.

In order to compare the immune states of acute phase and recovery phase of COVID-19 patients, we recruited COVID-19 patients in the early outbreak of the pandemic, collected the consecutive PBMC samples, and compared their immune profiles before and after negative conversion of SARS-CoV-2. In this analysis, immune cells tended to rest in late phase. We further analyzed the crosstalk between immune cells and elucidated the communication pattern and signaling transduction among them. Notably, IL-1, IL-8, and IGF1 signals, and CCL and CXCL signals, are attenuated at late phase,. In sum, features of communication pattern among monocytes, T cells, and B cells might be key indicators of recovery from COVID-19.

## Materials and Methods

### Sample Collection and Ethics Statement

Peripheral blood was obtained from three COVID-19 patients (C1, C2, and C3) with moderate symptoms during their early and late phases of disease progression, respectively. For patient C1, samples were collected in days 12 and 24 (days post reported symptom onset); for patient C2, samples were collected in days 8 and 19; and for patient C3, samples were collected in days 16 and 25. The demographic and clinical features were shown in the supplemental information (**Supplementary Figure S1A**). Sera were collected from six COVID-19 patients C1, C2, C3, C4, C5, and C6. This study was approved by the Institutional Review Board of the Institute of Microbiology, Chinese Academy of Sciences and Fifth Medical Center of Chinese PLA General Hospital. The study is compliant with all relevant ethical regulations regarding research involving human participants, and written informed consent was provided by all patients or legal representatives for participation.

Peripheral blood mononuclear cells (PBMCs) were then isolated and frozen in liquid nitrogen before use. In brief, anticoagulated blood was diluted with equal size of phosphate-buffered saline (PBS) and then added on top of Ficoll (Sigma Aldrich, St Louis, USA) carefully without disturbing the layer. After centrifuging at 600×*g* for 30 min, buffy coat was collected as PBMCs and washed with PBS twice. PBMCs were frozen with 90% fetal bovine serum (FBS) and 10% dimethyl sulfoxide (DMSO) using a Nalgene freezing container in −80°C freezer for more than 4 h and then transferred into liquid nitrogen.

### 10x Genomics Single-Cell Sequencing

PBMCs were thawed at 37°C and washed with cold medium [Roswell Park Memorial Institute (RPMI)-1640 with 10% fetal bovine serum] for subsequent experiments. Cells were resuspended in PBS and incubated with maglev magnetic beads (QDSphere, Beijing, China) to excluded dead cells. Afterward, cells were suspended in PBS with 0.04% BSA, passed through a 70-mm cell strainer (Thermo Scientific, Waltham, USA), and counted with Luna™ automated cell counter (Logos Biosystems, Anyang-si, South Korea). Cell counts were adjusted and loaded with 20,000 cells in the Chromium™ Controller for partitioning single cells into nanoliter-scale gel bead-in-emulsions (GEMs). Single Cell 3′ reagent kit v3 was used for reverse transcription, cDNA amplification, and library construction of the gene expression libraries following the detailed protocol provided by 10x Genomics (Chromium Single Cell 3′ Reagent Kits v3, Pleasanton, USA). Sequencing was performed using Hiseq XTen PE150 sequencer (Illumina, San Diego, USA).

### Single-Cell RNA-seq Data Processing and Analysis

Raw sequencing data were first subjected to quality check using FastQC and then trimmed with FASTX-Toolkit. Gene counts were calculated through aligning fastq reads to human GRCh38 genome using CellRanger 4.0. Single-cell clusters were annotated by their signature genes in Loupe Browser (10x Genomics). Unique molecular identifiers (UMIs) belonging to monocyte clusters were isolated and subjected to further analysis using Seurat 3.2. Poor-quality cells and doublets were removed based on UMI numbers and mitochondrial gene percentage in accordance with the following standards: gene expressed in <3 cells; genes inside one cell <200 ones; percentage of mitochondrial genes above 20%; and gene numbers per cell more than 15, 500, or <20. Variable genes were calculated using the Seurat function “FindVariableGenes” with the following criteria: x cutoff ranging from 0.05 to 8 and y cutoff of 0.5. A total of 2,973 variable genes among monocyte clusters were imported into the Seurat “RunPCA” pipeline, followed by non-linear dimensional reduction of the top 12 PCs using the Seurat function “RunTSNE”. Seurat function “FindClusters” was used to find clusters with resolution of 0.5. Scaled data were used for heatmap plotting, and normalized data were used for drawing violin plots. For feature plots, normalized data were plotted on the scatter plots of tSNE_1 and tSNE_2 in the form of pot colors. After excluding low-quality cells and erythrocytes, we processed and integrated scRNA-seq data and generated single-cell gene information of 23,839 cells with the average of 3,973 cells per sample.

### Differential Gene Expression Analysis

Differential gene expression analysis was performed using FindMarkers functions in Seurat V3. Genes expression with absolute value of fold change no <2 and adjusted p-value <0.05 were defined as significantly differentially expressed genes (DEGs).

### GO Enrichment Analysis

Significant DEGs between different clusters were abstracted as up- and downregulated genes and taken for GO enrichment analysis using clusterProfiler V3.16.1, respectively. Top 15 biological processes genes related were displayed as shown.

### Gene Set Enrichment Analysis

Gene set enrichment analysis were performed with chips generated by GSEA V4.1.0. Hallmark gene sets and Kyoto Encyclopedia of Genes and Genomes (KEGG) gene sets were chosen to run the analysis. Gene sets that met Normalized Enrichment Score (NES) >1, p-value <0.05, and false discovery rate (FDR) q-value <0.25 were defined as significant enrichment gene sets.

### Cell–Cell Communication Analysis

Cellular communication networks were quantitatively inferred and analyzed using scRNA-seq data. An open source R package CellChat was installed to visualize the communications among different cell groups (17). Two hundred twenty-nine signaling pathway families were grouped as a library to analyze cell–cell communication. Circle, hierarchy, and river plots were generated according to the ligand–receptor interaction network.

### Cytokines Array Assay

Quantitative cytokines/chemokines analysis was performed using 12 serum samples from COVID-19 patients (Quantibody Human Angiogenesis Array 1000, Raybiotech, Peachtree Corners, USA). Samples were diluted three times and incubated at room temperature for 1 h. After washing with Wellwash Versa Microplate Washer (Thermo Scientific, Waltham, USA), the slides were incubated with detection antibody for 2 h. Then, Cy3 equivalent dye-conjugated streptavidin were added into wells and incubated for 1 h. After washing and drying, fluorescence of the slides was detected with InnoScan 300 Microarray Scanner (Innopsys, Carbonne, France). Data were normalized and analyzed and then presented *via* GraphPad Prism 7 (GraphPad Software, Inc., La Jolla, CA).

### Data Availability Statement

The single-cell sequencing raw datasets in this study can be found in National Microbiology Data Center (https://nmdc.cn/), and the download IDs were NMDC20021910, NMDC20021911, NMDC20021912, NMDC20021913, NMDC20021914, and NMDC20021915.

## Results

### The Immune Profile of Peripheral Blood in COVID-19 Patients

**Figure 1:**
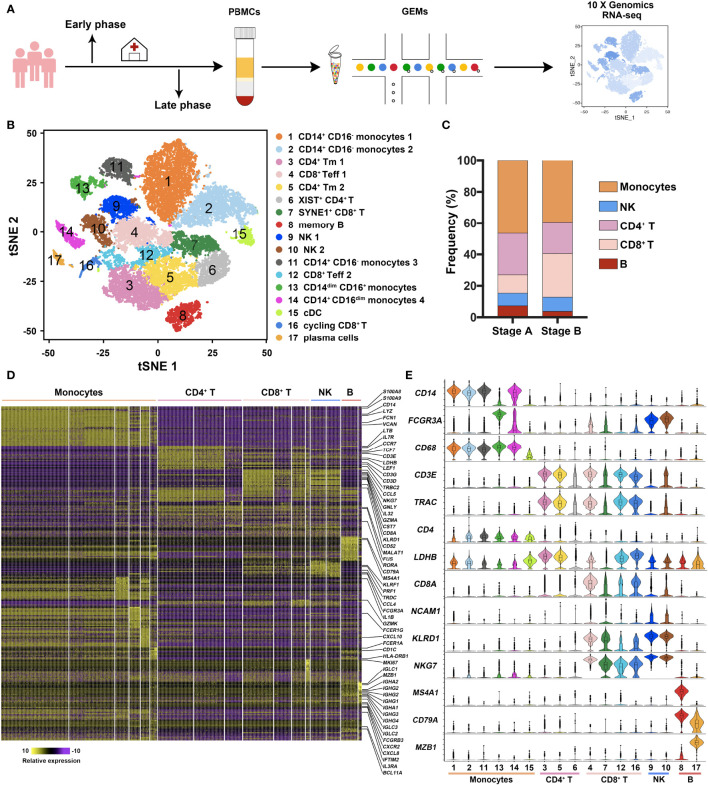
Study design and immune profile of COVID-19 patients. **(A)** Schematic depicting the overall design of the study. Three moderate COVID-19 patients (C1, C2, and C3) were recruited, and peripheral blood was collected twice during hospital admission. The first peripheral blood samples collected immediately after admission indicated as the early phase sample. After the nucleic acid detection of SARS-CoV-2 converted negative, the second sample (late phase) was collected. Peripheral blood mononuclear cells (PBMCs) were isolated and prepared into nanoliter-scale Gel Bead-In-Emulsions (GEMs) with 10× genomics Chromium Single Cell 3′ Reagent Kits. After reverse transcription, cDNA amplification, and library construction, gene expression libraries were sequenced and analyzed. **(B)** tSNE representation of immune cell clusters identified in sc-RNA seq data. Data of all six samples (two samples each patient) were pooled and analyzed. **(C)** Frequency distributions of five cell types of the three patients in in both stages A and B. Monocytes, CD4^+^T cells, CD8^+^T cells, NK cell, and B cells were represented as orange, medium lavender magenta, misty rose, blue, and brick red, respectively. **(D)** Heatmap of gene expression in five major immune cell subsets. Representative genes with high expression were listed beside. The relative expression level was defined from 10 to −10 and colored from yellow to purple. The higher the gene was expressed, the yellower the color was represented. **(E)** Expressions of representative genes in each subset were exhibited as violin plot.

To explore the immune profile of acute and recovery phases of COVID-19 patients, three moderate patients were recruited, and the first peripheral blood samples were collected immediately after admission. After the negative conversion of SARS-CoV-2 RNA, the second samples were collected (**Supplementary Figure S1A, B**). PBMCs attained at early phase (Stage A) and late phase (Stage B) were performed for single-cell RNA sequencing (**Figure 1A**). Seventeen clusters were identified and classified into five main immune cell subsets—myeloid, CD4^+^T, CD8^+^T, NK, and B cells (**Figure 1B**). Although the compositions of immune cells exhibited heterogeneity in individual sample, they showed similar change from early to late stage. Myeloid and B cells decreased apparently, while T cells augmented in stage B (**Figure 1C**).

In detail, six clusters were identified as monocytes—CD14^+^CD16^−^ monocytes 1 (Cluster 1-S100A8^+^, S100A9^+^), CD14^+^CD16^−^ monocytes 2 (Cluster 2-LYZ^+^, VCAN^+^), CD14^+^CD16^−^ monocytes 3 (Cluster 11-IL1B^+^, HLA-DRB^+^), CD14^dim^CD16^+^ monocytes (Cluster 13-FCGR3A^+^CDKN1C^+^), CD14^+^CD16^dim^ monocytes 4 (Cluster 14-IFI27^+^TYMS^+^), and cDCs (Cluster 15-FCER1A^+^CD1C^+^). Three clusters were defined as CD4^+^T cells, i.e., memory CD4^+^T cells 1 (Cluster 3-LTB^+^, IL7R^+^, CCR7^+^), memory CD4^+^T cells 2 (Cluster 5-LTB^+^, IL7R^+^, CCR7^+^), and XIST^+^CD4^+^T cells (Cluster 6-XIST^+^, BCL11B^+^, MALAT1^+^, FUS^+^). CD8^+^T cells were composed of effector CD8^+^T cells 1 (Cluster 4-CCL5^+^, GZMH^+^, GNLY^+^), SYNE1^+^CD8^+^T cells (Cluster 7-SYNE1^+^, SYNE2^+^, XIST^+^), effector CD8^+^T cells 2 (Cluster 12-GZMK^+^, IL32^+^, IL7R^+^), and cycling CD8^+^T cells (Cluster 16-MKI67^+^, TMYS^+^, STMN1^+^). Clusters 9 and 10 (GNLY^+^, NKG7^+^, KLRD1^+^) were identified as NK cells. B cells included memory B cells (Cluster 8-MS4A1^+^, CD79A^+^, IGHD^+^) and plasma cells (Cluster 17-MZB1^+^, SDC1^+^). Heatmap was generated according to the top 20 genes of each cluster (**Figure 1D**), while feature genes were listed to show the overall view of these immune cells (**Figure 1E**).

### Dynamic Features of Monocytes During Moderate COVID-19 Progression

**Figure 2:**
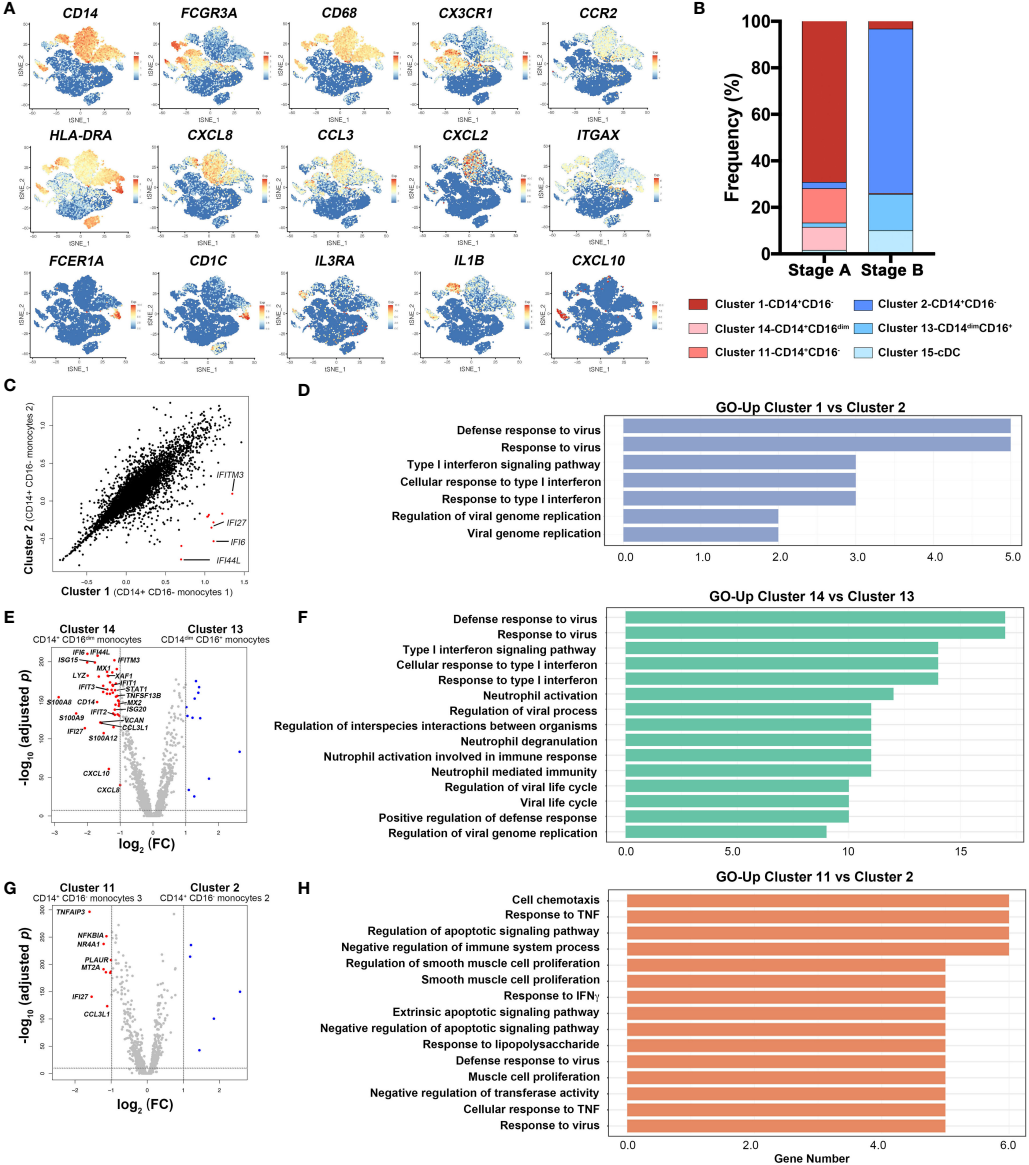
Dynamic features of monocytes in COVID-19 patients. **(A)** Feature plots of characteristic genes were represented *via* tSNE. The relative gene expression is shown from blue to red. **(B)** Frequency distribution of six myeloid cell clusters, i.e., Cluster 1 (CD14^+^CD16^−^ monocytes 1), Cluster 2 (CD14^+^CD16^−^ monocytes 2), Cluster 11 (CD14^+^CD16^-^ monocytes 3), Cluster 13 (CD14^dim^CD16^+^ monocytes), Cluster 14 (CD14^+^CD16^dim^ monocytes 4), and Cluster 15 (cDCs), in each stage. **(C)** Scatter plot of genes expression in Cluster 1 (CD14^+^CD16^−^ monocytes 1) and Cluster 2 (CD14^+^CD16^−^ monocytes 2). Differentially expressed genes in Cluster 1 are highlighted as red dots. **(D)**. GO analysis with genes highly expressed in Cluster 1 comparing with Cluster 2. **(E)** Volcano plots of Cluster 14 (CD14^+^CD16^dim^ monocytes) versus Cluster 13 (CD14^dim^CD16^+^ monocytes). **(F)** GO analysis with genes highly expressed in Cluster 14 comparing with Cluster 13. **(G)** Volcano plots of Cluster 11 (CD14^+^CD16^−^ monocytes 3) versus Cluster 2 (CD14^+^CD16^−^ monocytes 2). **(H)** GO analysis with genes highly expressed in Cluster 11 comparing with Cluster 2. Differential expressed genes were defined with threshold of fold change ≥2 and p-value <0.05 and represented as red or blue.

To further analyze the features of monocytes, we performed feature plots using relative genes (**Figure 2A**). Classical CD14^+^CD16^−^ monocytes (Cluster 1, 2, 11, 14) CD68^+^, CD14^+^, and FCGR3A^−^; non-classical CD14^−^CD16^+^ monocytes (Cluster 13) CD68^+^, CD14^−^, FCGR3A^+^, and IL3RA^+^; cDCs (Cluster 15) FCGR1A^+^, CD1C^+^, ITGAX^+^, and IL3RA^low^ were identified according to featured markers, among which CD14^+^CD16^−^ monocytes expressed CXCL8, CCL3, CX3CR1, and CCR2. CD14^+^CD16^−^ monocytes 3 (Cluster 11) also secreted IL1β and CD14^+^CD16^dim^ monocytes 4 (Cluster 14) highly expressed CXCL10, indicating their inflammatory function (18). We calculated frequencies of the six clusters and found that inflammatory monocytes (Clusters 1, 11, and 14) comprised more than 80% of monocytes in stage A. Notably, proportions of Clusters 2, 13, and 15 that were less inflammatory (termed regulatory monocytes) scaled up in stage B (**Figure 2B**).

Subsequently, we compared differentially expressed genes (DEGs) of CD14^+^CD16^−^ monocyte subsets (Clusters 1 and 2) in two stages. Cluster 1 is abundant in stage A, and Cluster 2 is the major monocyte in stage B. It turned out that Cluster 1 expressed high levels of interferon-inducible genes such as IFITM3, IFI27, IFI6, and IFI44L, which played important roles in inflammation and type I interferon response (**Figure 2C**). GO analysis showed that genes upregulated in Cluster 1 were related to virus defense and type I interferon response comparing with Cluster 2 (**Figure 2D**). As mentioned previously, monocytes in stage A were mainly comprised of CD14^+^CD16^−^ monocytes 1 (Cluster 1), CD14^+^CD16^−^ monocytes 3 (Cluster 11), and CD14^+^CD16^dim^ monocytes 4 (Cluster 14), while in stage B, CD14^+^CD16^−^ monocytes 2 (Cluster 2) and CD14^−^CD16^+^ monocytes (Cluster 13) were the primary subsets (**Figure 2B**). To further compare the features of monocytes in stages A and B, we analyzed DEGs between CD14^+^CD16^dim^ monocytes 4 (Cluster 14) and CD14^−^CD16^+^ monocytes (Cluster 13), and CD14^+^CD16^−^ monocytes 3 (Cluster 11) and CD14^+^CD16^−^ monocytes 2 (Cluster 2), respectively (**Figures 2E, G**). Inflammatory monocytes (Cluster 14) cells stage A subset were highly activated, expressing a bunch of ISGs (IFI6, IFI44L, IFITM3, IFIT1, IFIT2, IFI27, ISG15, ISG20, MX1, and MX2), inflammation-related genes (S100A8, A100A9, and S100A12), and chemokines (CXCL10, CXCL8, and CCL3L1) (**Figure 2E**). Another stage A subset of inflammatory monocyte (Cluster 11) highly expressed inflammatory-related gene (TNFAIP3) and ISG (IFI27), and genes involved in inhibition of apoptosis process (NFKBIA) (**Figure 2G**). We further performed GO analysis between inflammatory monocytes and regulatory monocytes (Cluster 14 vs. 13, Cluster 11 vs. 2) (**Figures 2F, H**). Genes related with defense response to virus and type I interferon response were enriched in Cluster 14 (stage A subset). In addition, genes related with cell chemotaxis and response to virus and cytokines were augmented in Cluster 11 (stage A subset) comparing with Cluster 2 (stage B subset). Besides, gene set enrichment analysis (GSEA) was performed in inflammatory subsets (Clusters 1, 11, and 14) and regulatory subsets (Clusters 2 and 13). Genes of CD14^+^CD16^−^ monocytes 3 (Cluster 11) enriched in mTORC1 signaling pathway, while CD14^dim^CD16^+^ monocytes (Cluster 13) expressed more genes associated with toll-like receptor signaling pathway (**Supplementary Figures S2A, B**). In sum, expressions of genes involved in type I interferon signaling pathway, virus response, monocyte activation, and cell chemotaxis were higher in stage A, indicating a hyperactive inflammatory response and antiviral procession in early phase in moderate COVID-19 patients.

### Activation of T Cells Dampened in Recovery Phase

**Figure 3:**
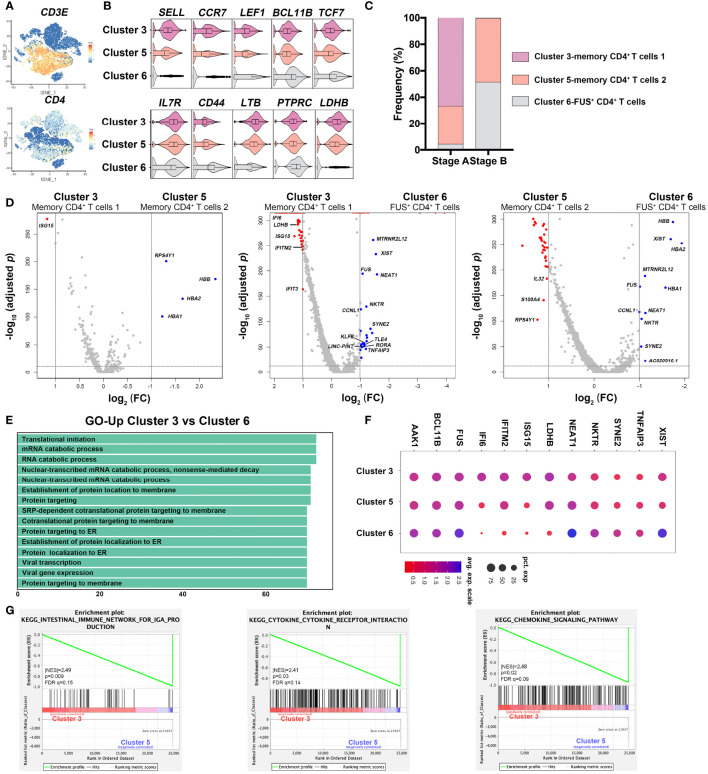
Characterization of CD4^+^T cells in different stages in COVID-19 patients. **(A)** Feature plots of characteristic genes of CD4^+^T cells were represented *via* tSNE. **(B)** Genotypes of three CD4^+^T cell subsets, namely, Cluster 3 (memory CD4^+^T cells 1), Cluster 5 (memory CD4^+^T cells 2), Cluster 6 (XIST^+^CD4^+^T cells). **(C)** Frequency distribution of the three CD4^+^T cell clusters in each stage. **(D)** Volcano plots of Cluster 3 (memory CD4^+^T cells 1) versus Cluster 5 (memory CD4^+^T cells 2), Cluster 3 (memory CD4^+^T cells 1) versus Cluster 6 (XIST^+^CD4^+^T cells), and Cluster 5 (memory CD4^+^T cells 2) versus Cluster 6 (XIST^+^CD4^+^T cells). Differentially expressed genes were defined with threshold of fold change ≥2 and p-value <0.05 and represented as red or blue. **(E)** GO analysis with genes highly expressed in Cluster 3 comparing with Cluster 6. **(F)** Signature genes generated from differential gene expression analysis. Size and color of dots are related with counts of gene expression. Bigger and bluer dots represent higher gene expression. **(G)** Gene sets enrichment analysis (GSEA) between Cluster 3 (memory CD4^+^T cells 1) and Cluster 5 (memory CD4^+^T cells 2). Cluster 5 had genes enriched in “intestinal immune for IgA production,” “cytokine–cytokine receptor interaction,” and “chemokine signaling pathway.” Significant enrichment was defined by |NES| > 1, NOM p-val < 0.05, FDR q < 0.25.

According to the scRNA-sequencing data, three clusters were identified as CD4^+^T cells (**Figure 3A**). Unlike memory CD4^+^T cells (Clusters 3 and 5), XIST^+^CD4^+^T cells (Cluster 6) expressed lower levels of SELL, CCR7, LEF1, LTB, LDHB, and TCF7 and higher levels of BCL11B and IL7R (**Figure 3B**). CD4^+^ memory T cells were dominant T cells in stage A; however, XIST^+^CD4^+^T cells were significantly increased in stage B (**Figure 3C**). We further compared features among the three clusters. Memory CD4^+^T cells 1 and 2 shared similar expression profile. Interestingly, they highly expressed ribosome synthesis genes and IFN-induced genes comparing with XIST^+^CD4^+^T cells (Cluster 6) (**Figures 3D, E**), indicating that levels of protein synthesis and interferon response were more activated in stage A. Similarly, GO analysis showed CD4^+^T-cell-enriched processes such as protein targeting, establishment of protein location to membrane, and translational initiation in stage A (**Supplementary Figure S2C**). Meanwhile, IFI6, IFITM2, ISG15, and LDHB were highly expressed in memory CD4^+^T cells, which were crucial for cell hypermetabolism and defense against virus infection. On the other hand, XIST^+^CD4^+^T cells were considered as a functional subset with high expression of genes associated with survival and differentiation (19). We found that the expression level of XIST, BCL11B, NEAT1, AAK1, FUS, and TNFAIP3 genes were high in XIST^+^CD4^+^T cells (**Figure 3F**), indicating the maturation of T cells (20–23). GSEA analysis showed that genes related with IgA production, cytokine–receptor interaction, and chemokine signaling pathway were enriched in memory T cells (**Figure 3G**). Thus, immune responses of CD4^+^T cells were hyper-inflammatory and activated in early phase of COVID-19, and they underwent functional change during recovery process.

Four clusters were identified as CD8^+^T cells with the expressions of signature genes (**Supplementary Figure S3A**). Distribution of these subsets were analyzed (**Supplementary Figure S3B**). SYNE1^+^CD8^+^T cells (Cluster 7) increased while cycling CD8^+^T cells (Cluster 16) decreased in stage B. Cycling CD8^+^T (Cluster 16) cells showed cytotoxic capacity with high expression level of Granzyme A (GZMA), indicating their function in early phase of in virus defense. We further analyzed the signature of SYNE1^+^CD8^+^T (Cluster 7) cells that were increased in recovery stage. Unlike classical effector CD8^+^T cells, SYNE1^+^CD8^+^T cells (Cluster 7) expressed low levels of granzyme and ISGs (**Supplementary Figure S3C**), indicating the weakened activation. Two subsets of effector CD8^+^T cells were analyzed. Effector CD8^+^T cells 1 (Cluster 4) highly expressed GZMB, GNLY, FGFBP2, and KLRD1, while effector CD8^+^T cells 2 (Cluster 12) expressed high levels of GZMK, IL7R, and LTB (**Supplementary Figure S3C**). To further elucidate the function of major CD8^+^T subsets in stages A and B, we compared the differential gene expression of the two effector CD8^+^T cells (Clusters 4 and 12) with SYNE1^+^CD8^+^T cells (Cluster 7) respectively. The major CD8^+^T cells in stage A, including effector CD8^+^T cells 1 (Cluster 4), effector CD8^+^T cells 2 (Cluster 12), and cycling CD8^+^T cells (Cluster 16), exhibited higher expression levels of S100A6, S100A10, S100A11, RPS26, and GZMA. However, SYNE1^+^CD8^+^T cells (Cluster 7), which was increased in stage B, expressed high levels of SYNE1, SYNE2, XIST, and KLRK1 (**Supplementary Figure S3E**) and may play a role in the elimination of virus-infected cells and formation of memory T cells (24, 25). GO analysis showed genes mainly involved in processes such as “protein targeting to ER” and “translational initiation”, suggesting high rate of protein synthesis in effector CD8^+^T cells (**Supplementary Figure S3F**). Taken together, CD8^+^T cells highly expressed granzyme-related genes and were more cytotoxic in early phase of COVID-19. Both CD4^+^T and CD8^+^T cells were activated and inflammatory in early phase (stage A) and accumulated mature and memory T cells during convalescent process (stage B), which were critical for recovery from COVID-19.

### Immune Features of NK and B Cells

There were two clusters identified as NK cell, with expression of NCAM1 and NCR1 (**Supplementary Figure S4A**). As NK cells were majorly clustered as Cluster 10 in stage A and Cluster 9 in stage B (**Supplementary Figure S4B**), we analyzed DEGs between the two subsets. In stage A, NK cells expressed high levels of XIST, IFI6, IFI44L, IFIT3, ISG15, MX1, XAF1, and CCL3, which were not observed in stage B (**Supplementary Figure S4C**). GO analysis showed that NK cells in stage A were augmented in type I interferon responses and anti-virus responses, indicating an activated status of NK cells in early phase of COVID-19 (**Supplementary Figure S4D**).

We also identified two clusters as B cells, i.e., memory B cells (Cluster 8) and plasma cells (Cluster 17) (**Supplementary Figure S4A**). A large proportion of memory B cells were found in both stages A and B (**Supplementary Figure S4G**). Memory B cells expressed CD19 and CD20 extra, and plasma cells were featured by CD38 and MZB1 (**Supplementary Figure S4F**). DEGs analysis showed that memory B cells were enriched in ribosome-related genes, indicating an active status. We further measured levels of Ig immunoglobulin-related genes and figured out that IgM was mainly expressed by memory B cells, while IgA1 and IgGs were mainly secreted by plasma cells (**Supplementary Figure S4E**). Moreover, memory B cells expressed a high level of HLA-DRA, indicating their close communications with CD4^+^T cells.

### Cell–Cell Contact Communications Between Different Immune Subsets

**Figure 4:**
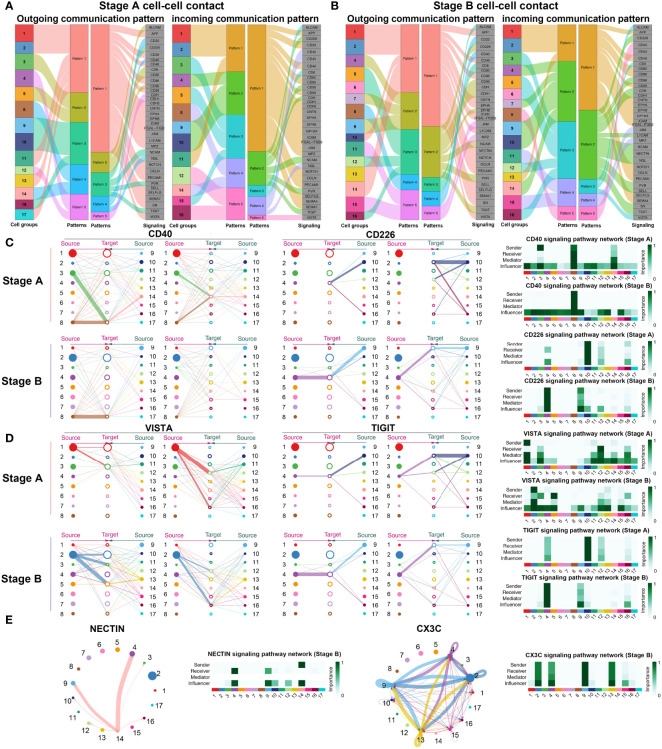
Cell–cell contact communications among immune cells. **(A)** Outgoing and incoming communication patterns of cell–cell contact signaling pathways in stage A. Outgoing and incoming communication patterns of cell–cell contact signaling pathways clustered into five patterns as indicated in the heatmap. In outgoing communication patterns, Clusters 1–17 are indicated as different colors and classified into five patterns (patterns 1–5) except Clusters 6, 7, and 15, according to the outgoing signals they shared. Pattern 1 includes Cluster 1, Cluster 2 (CD14^+^CD16^−^ monocytes), Cluster 11 (CD14^+^CD16^−^ monocytes), Cluster 13 (CD14^dim^CD16^+^ monocytes), and Cluster 17 (plasma cells). Pattern 2 includes Cluster 3 (CD4^+^Tm) and Cluster 5 (CD4^+^Tm). Pattern 3 includes Cluster 9 (NK), Cluster 10 (NK), and Cluster 16 (cycling CD8^+^T). Pattern 4 includes Cluster 8 (memory B) and Cluster 14 (CD14^+^CD16^dim^ monocytes). Pattern 5 includes Cluster 4 (CD8^+^Teff) and Cluster 12 (CD8^+^Teff). In incoming communication patterns, Clusters 1–17 were classified into five patterns (patterns 1–5) except for Clusters 6, 7, 13, and 17. Pattern 1 includes Clusters 1, Cluster 2 (CD14^+^CD16^−^ monocytes), and Cluster 11 (CD14^+^CD16^−^ monocytes). Pattern 2 includes Cluster 4 (CD8^+^Teff), Cluster 9 (NK), and Cluster 10 (NK). Pattern 3 includes Cluster 3 (CD4^+^Tm), Cluster 5 (CD4^+^Tm), and Cluster 15 (cDCs). Pattern 4 includes Cluster 8 (memory B) and Cluster 14 (CD14^+^CD16^dim^ monocytes). Pattern 5 includes Cluster 12 (CD8^+^Teff) and Cluster 16 (cycling CD8^+^T). Signals included in these patterns are indicated in gray boxes. **(B)** Outgoing and incoming communication patterns of cell–cell contact signaling pathways in stage B. Outgoing and incoming communication patterns of cell–cell contact signaling pathways clustered into five patterns as indicated in the heatmap. **(C)** Hierarchical network of activated signals in stages A, B. **(D)** Hierarchical network of inhibitory signals in stages A, B. Signals sent by specific cluster are drawn with the same color indicated in **(A)**. Source and target of signaling pathways are connected by lines. Thickness of lines indicate the degree of the signal. Sender, receiver, mediator, and influencer of specific signals are indicated in individual heatmap. **(E)** Signals detected uniquely in stage B. Thickness of lines indicate the degree of the signal. Sender, receiver, mediator, and influencer of specific signals are indicated in individual heatmap.

As comparing the differential expressed genes may not reflect the signaling network *via* soluble and membrane-bound factors, we analyzed the cellular communications among immune cells in stages A and B, respectively, by using a newly developed method that curated a comprehensive signaling molecule interaction database (26).^17^ We performed cell–cell contact analysis of cells in both stages. Outgoing and incoming communication patterns were concluded in stages A and B to comprehend function network of immune cells (**Figure 4** and **Supplementary Figures S3B, S5A**).

Notably, in stage A, CD8^+^T cells and NK cells tended to send signals of CD226, NCAM, PVR, and TIGIT (pattern 3—outgoing) and CDH and CDH1 (pattern 5—outgoing), and received signals of CD226, CD96, CDH, NCAM, PVR, and TIGIT (pattern 2—incoming), and VISTA (pattern 5—incoming) (**Figure 4A** and **Supplementary Figure S5A**). Inhibitory signals in effector and SYNE1^+^CD8^+^T cells (Clusters 4 and 7) and NK cells were abundant in stage B (**Figure 4B** and **Supplementary Figure S5B**). Activated communication signals *via* cell–cell contact were analyzed in stages (**Figure 4C**). CD4^+^T cells communicated with memory B cells through CD40LG-CD40 in stage A but not in stage B. For inhibitory signals, monocytes transmitted VISTA signals to CD8^+^T cells (Clusters 12 and 16) in stage A but majorly to CD4^+^T cells (Clusters 3 and 5) and a bunch of CD8^+^T cells in stage B (**Figure 4D**), revealing a different regulatory pattern in the two stages. Source and target of TIGIT signal changed from NK cells (Cluster 10) to CD8^+^T cells (Cluster 4) in stage B, indicating that one inhibitory signal might regulate various subsets during the disease progression.

In addition, there were extra signals detected in stage B, such as NECTIN and CX3CL signals (**Figure 4E** and **Supplementary Figure S6C**). For NECTIN signal, monocytes (Cluster 14) communicated with CD8^+^T cells and NK cells through interaction with CD226 and TIGIT exerting an inhibitory role. Besides, CD8^+^T cells (Cluster 4), NK cells (Cluster 9), and regulatory monocytes (Clusters 2 and 13) interacted with each other *via* CX3CL1–CX3CR1 interaction in stage B, which was not observed in stage A (**Figure 4E** and **Supplementary Figure S6C**). The crosstalk between NK/T cells and non-classical (regulatory) CD14^dim^CD16^+^ monocytes *via* CX3CL1–CX3CR1 might be a feature of recovery phase in SARS-CoV-2 infection. In sum, the cell contact of immune cells varies in different phase of COVID-19, and several patterns of cell–cell contact might indicate the status of disease progression.

### Secreted Signaling Reveals an Altered Signal Transduction of IGF in Recovery Phase

**Figure 5:**
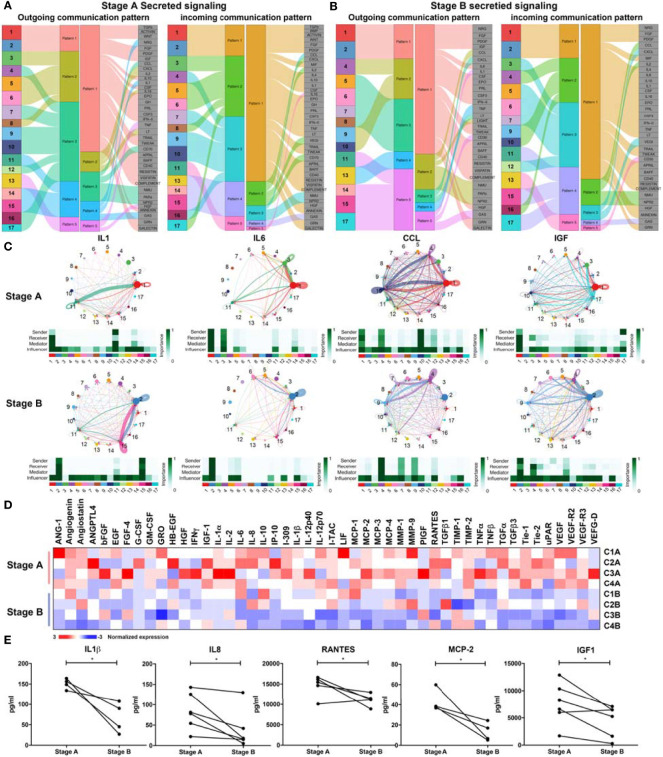
Secreted signaling communications among immune cells. **(A)** Outgoing and incoming communication patterns of secreted signaling pathways in stage A. In outgoing communication patterns, Clusters 1–17 are indicated as different colors and classified into five patterns (patterns 1–5), according to the outgoing signals they shared. Pattern 1 includes Clusters 1 and 2 (CD14^+^CD16^-^ monocytes). Pattern 2 includes Cluster 3 (CD4^+^Tm), Cluster 5 (CD4^+^Tm), Cluster 6 (XIST^+^CD4^+^T), and Cluster 8 (memory B). Pattern 3 includes Cluster 4 (CD8^+^Teff), Cluster 7 (SYNE1^+^CD8^+^T), Cluster 9 (NK), Cluster 10 (NK), Cluster 12 (CD8^+^Teff), Cluster 16 (cycling CD8^+^T), and Cluster 17 (plasma cells). Pattern 4 includes Cluster 11 (CD14^+^CD16^-^ monocytes), Cluster 14 (CD14^+^CD16^dim^ monocytes), and Cluster 15 (cDCs). Pattern 5 includes Cluster 13 (CD14^dim^CD16^+^ monocytes). In incoming communication patterns, Clusters 1–17 were classified into five patterns (patterns 1–5) except for Cluster 12. Pattern 1 includes Clusters 1 and 2 (CD14^+^CD16^-^ monocytes). Pattern 2 includes Cluster 3 (CD4^+^Tm), Cluster 5 (CD4^+^Tm), Cluster 6 (XIST^+^CD4^+^T), Cluster 8 (memory B), and Cluster 17 (plasma cells). Pattern 3 includes Cluster 4 (CD8^+^Teff), Cluster 7 (SYNE1^+^CD8^+^T), Cluster 9 (NK), Cluster 10 (NK), and Cluster 16 (cycling CD8^+^T). Pattern 4 includes Cluster 13 (CD14^dim^CD16^+^ monocytes), Cluster 14 (CD14^+^CD16^dim^ monocytes), and Cluster 15 (cDCs). Pattern 5 includes Cluster 11 (CD14^+^CD16^-^ monocytes). Signals included in these patterns are indicated in gray boxes. **(B)** Outgoing and incoming communication patterns of secreted signaling pathways in stage B. Outgoing and incoming communication patterns also clustered to five patterns, respectively. **(C)** Communication network of secreting signals in stage A and stage B. Signals sent by specific cluster are drawn with the same color indicated in panel **(A)** Target of signaling pathways are pointed by line arrows. Thickness of lines indicate the degree of the signal. Sender, receiver, mediator, and influencer of specific signals are indicated in individual heatmap. **(D)** Levels of 48 angiogenic proteins/cytokines/chemokines in stages A and B are generalized and presented in the heatmap. Serum samples from four moderate patients are used and analyzed as shown. The normalized expression is scaled from −3 to 3. **(E)** Specific inflammatory factors (IL1β, IL8, RANTES, MCP-2, and IGF1) were significantly decreased in stage B (n = 6 in IGF1, IL8, RANTES; n = 4 in MCP2, IL1β, IP-10). Paired t-test was performed, and significances were inferred as p < 0.05 (*).

Secreted signaling pathways were analyzed and classified into five patterns (**Figures 5A, B**). Compared with stage A, the communication patterns changed a lot in stage B (**Figure 5B** and **Supplementary Figures S3D, S5C**). Intriguingly, for stage A, CD4^+^T cells (Clusters 3, 5, and 6) and memory B cells (Cluster 8) tended to transduce signals through the same pattern, which included CXCL, IL2, IL16, and LT signaling pathways and received signals from CXCL, IL2, VEGI, and CD70 signaling pathways (**Figure 5A** and **Supplementary Figure S5C**). SYNE1^+^CD8^+^T cells joined the communication network in stage B (**Figure 5B** and **Supplementary Figure S5D**). Different from lymphocytes, monocytes presented variable communication patterns (patterns 1, 4, and 5—outgoing and incoming), e.g., TGFβ, IGF, IL10, TRAIL, and CD40 in stage A (**Figure 5A**). In stage B, monocytes also involved in similar signals with those in stage A, including IL1 and GAS, with several differences in patterns of signals of such as IL10 and IL6, which were vital in regulating T cells (**Figure 5B**).

To further compare the communication patterns of stages A and B, signals of cytokines were analyzed. Signals that promote activation of immune cells (IL1, IL-4, IL6, PARs, MIF, and LT) were apparently changed (**Figure 5C** and **Supplementary Figure S7A**). The inflammatory signals (IL-1, IL-6, and CCL) were attenuated in stage B. Meanwhile, the sender of inflammatory signals (IL1, IL-4, IL6, PARs, MIF, and LT) changed from inflammatory subsets to regulatory or rest subsets (**Figure 5C** and **Supplementary Figure S7A**). In stage A, intense CCL signals including CCL5-CCR1/3/5 and CCL3-CCR1/5 were observed among effector CD8^+^T cells (Cluster 4), NK cells (Cluster 10), and monocytes (Cluster 1), while the network changed to effector CD8^+^T cells (Cluster 4), CD8^+^T cells (Cluster 7), and NK cells (Cluster 9). Meanwhile, the contribution of CCL3-CCR1/5 was weakened in stage B, which indicated a less inflammatory status in stage B (**Figure 5C** and **Supplementary Figure S6A**).

Surprisingly, we found that metabolic signal and IGF signal were also changed in the recovery phase. The source of IGF signal switched from inflammatory monocytes (Cluster 1) and plasma cells (Cluster 17) to regulatory CD14^+^CD16^−^ monocyte (Cluster 2). IGF is involved in insulin receptor substrate (27)-initiated PI3K-AKT/mTOR pathway and was implicated in ARDS (28–30). The conversion of IGF signal might be associated with the recovery of COVID-19 patients. The role of IGF in progression of COVID-19 is worthy to be further analyzed.

Apart from activated signals, regulatory signals and chemokine signals were also detected among immune cells (**Supplementary Figure S7B**). LIGHT and TGFβ signals were involved in CD8^+^T cells (Cluster 4) in stage B, which might synergistically regulate cytotoxic function of CD8^+^T cells. Comparing with stage A, IL10 signal seemed enhanced in stage B, and effector CD8^+^T cells (Cluster 4) served as a main source (**Supplementary Figure S7B**). Although BTLA is an inhibitory molecule in T cells, it can transduce costimulatory signals through binding with HVEM (31, 32). Strong signals among CD4^+^T cells, memory B cells, and monocytes in stage A (**Supplementary Figure S7B**) indicate activation of BTLA in T and B cells in the early phase (33, 34), which was in concordance with a previous report that severe COVID-19 expressed higher BTLA (35).

Moreover, the composition of ligand–receptor pairs in several signaling pathways were consistent in stages A and B, including VISTA, IL6, IGF, CXCL, IL10, BTLA, and CD40 signaling pathways (**Supplementary Figure S6B**). Taken together, intense connections of activated and inflammatory signals were detected in stage A, whereas, inhibitory and regulatory signals were enriched in stage B.

### Downregulation of Serum Level of Inflammatory Cytokines and IGF1 in Recovery Patients

To further define immune status of COVID-19 patients, we analyzed levels of human angiogenic proteins in blood serums from patients with moderate symptoms. We found that inflammatory cytokines and several angiogenin-related factors were decreased in recovery phase (**Figure 5D**). Moderate patients showed higher levels of cytokines associated with inflammation and chemotaxis in stage A comparing with those in stage B (**Figures 5D, E**). Cytokines and chemokines including IL-12p40, IL-12p70, IL1β, TGFβ, TNF, CCLs (RANTES, MCP-1, MCP-2, MCP-3, MCP-4), and CXCLs (GRO, I-TAC, I-309, IL8, IP-10), and angiogenesis-related proteins (ANG-1, angiogenin, angiostatin, ANGPTL4, VEGF) were high in stage A comparing with those in stage B. Levels of IL1β, IL8, RNATES (CCL5), and MCP2 (CCL8) were significantly lower in stage B, and IP-10 (CXCL10), which had the similar trend (**Figures 5D, E**). These data were consistent with the conclusion from communication analysis (**Figure 5C**) and previous data (9, 36, 37). Notably, IGF1 showed significant decline during disease progression in all patients, which is consistent with our previous analysis (**Figure 5C**), indicating that IGF1 might contribute to the inflammatory status in stage A. The decreased levels of IGF1 in patient serum might be considered as an indicator of recovery (**Figure 5E**). In sum, downregulation of serum levels of inflammatory cytokines and IGF1 is associated with the recovery of COVID-19 patients.

## Discussion

Thousands of SARS-CoV-2 infections are being reported daily around the world. Although vaccination were developed, it is still urgent to explore the immune profile of COVID-19 patients and decipher the mechanism of how immune cells deal with the virus infection. In this study, we collected the PBMCs of moderate COVID-19 patients at acute and recovery phases and compared the dynamic distribution, characters, and communications of immune cell subsets between the two stages. Immune cells showed high expression of ISGs and calprotectin (S100A8 and S100A9) in stage A, which associated with more severe immune features and were concordant with previous reports (11, 38, 39). In cell communication analysis, T cells tended to transmit to a less inflammatory status in stage B. Activated signals (CD40 signal) that B cells received from CD4^+^T cells attenuated in stage B. Meanwhile, activated and inflammatory signals were abundant in stage A, such as IL1, CCL, MIF, and LT signals, while regulatory signals such as TGFβ and IL10 signals were enriched in stage B. We further verified our perspective by measuring cytokine/chemokines in serum *via* cytokine array assay. Decline of IL1β, IL-8, CCL5, and CCL8 in serum of patients revealed a cytokine signature during their disease progression. Of note, downregulation of IGF1 serum level was also associated with disease recovery. Taken together, immune cells presented high levels of type I interferon response and effector molecules in early phase of COVID-19 and turned inactive and less inflammatory along with decreasing in viral load. Our work provided the dynamic immune response and communications of immune cells during COVID-19 disease progression in the early SARS-CoV-2 pandemic.

Composition and communication patterns of immune cells changed from stage A to stage B. A decrease in monocytes and an increasing trend in T cells were observed, which might indicate a recovery progression. Although T cells were generally increased in stage B, their activation seemed to be downregulated in the late phase. XIST^+^CD4^+^T cells and SYNE1^+^SYNE2^+^CD8^+^T cells took a high occupancy in the later phase with less inflammatory features, which indicated a rest status comparing with that in early phase. All these transformations illustrated that activated signals for adaptive immune cells attenuated in the late phase, which might be signatures of recovery status.

Dysregulated immune features of COVID-19 patients have been reported in previous studies (11, 40–44). In accordance with previous works, ISGs, inflammatory genes, and effector molecules were high in stage A, indicating a highly effective and inflammatory status of immune cells. Besides, several cytokine levels evaluated with BALF reflected inflammatory status of the infection sites. Zaid et al. compared the cytokines in serum and BALF of severe COVID-19 patients and found that both of them contained significantly elevated levels of CXCL1, CXCL8, CXCL12, CCL2, CCL3, CCL5, EGF, VEGF, and PDGF-AB/BB (45). It is possible that the cytokines levels of BALF might reflect the inflammatory status of the pulmonary mucosal site. The immune cells and cytokines of peripheral blood might be related to the status of systemic immune system and could be indicators of the disease outcome. Several studies of longitudinal immune profiles were performed (46). Similarly, we observed decreases in IL6, IP-10, and MCP-1 in the assay of cytokine/chemokine measurement with plasma. What is more, we found out that the levels of IL1β, IL8, RANTES (CCL5), MCP-2, and IGF1 were significant higher in early stage of infection. As IL1β, IL8, RANTES, and MCP-2 were reported as important factors related in severe symptoms (9, 11, 47), their high levels might be related with the immunopathology in acute phase of mild/moderate infection. Moreover, the sender of inflammatory signals changed from inflammatory subsets to regulatory or rest subsets, which might be a feature of disease recovery.

However, there were few researches focusing on the relationship of IGF1 and COVID-19. In a previous research, IGF1 was evaluated in patients with ARDS (48). Here, we figured out a higher level of IGF1 in early stage of infection in COVID-19 patients. As the source of IGF1 was mainly from plasma B cells in stage A, it might be a key factor in regulating immune responses in early stage of infection. Further researches are required to confirm the mechanism of IGF1 in B cells regulating immune system in acute stage for defending against COVID-19 infection.

The analysis of immune profiles in early and late stages of COVID-19 patients who developed symptoms reveal that monocytes and lymphocytes turned less inflammatory in recovery phase. The patterns of cell communication together with serum soluble factors reflect the status of disease and could be considered as indicators of the progression of COVID-19.

## Data Availability Statement

The data has been deposited to the National Microbiology Data Center; you may find the data: https://nmdc.cn/resource/genomics/sra/detail/NMDC40008644. The accession number is NMDC4008644.

## Ethics Statement

The studies involving human participants were reviewed and approved by Institutional Review Board of the Institute of Microbiology, Chinese Academy of Sciences and Fifth Medical Center of Chinese PLA General Hospital. The patients/participants provided their written informed consent to participate in this study.

## Author Contributions

MZ designed and performed experiments, analyzed data, and wrote the paper. FS, WZ, and ZC performed experiments. PX and ZL analyzed data. PY provided patient samples and analyzed data. SW initiated the study and organized, designed, and wrote the paper. All authors contributed to the article and approved the submitted version.

## Conflict of Interest

The authors declare that the research was conducted in the absence of any commercial or financial relationships that could be construed as a potential conflict of interest.

## Publisher’s Note

All claims expressed in this article are solely those of the authors and do not necessarily represent those of their affiliated organizations, or those of the publisher, the editors and the reviewers. Any product that may be evaluated in this article, or claim that may be made by its manufacturer, is not guaranteed or endorsed by the publisher.
