# Supplementary material for: Communication Pattern Changes Along With Declined IGF1 of Immune Cells in COVID-19 Patients During Disease Progression
Source: Front Immunol. 2022 Jan 14;12:729990. doi: 10.3389/fimmu.2021.729990 (PMC8795624; doi:10.3389/fimmu.2021.729990)
Supplement: Supplementary file 1 [file DataSheet_1.docx]

**Supplemental Information**

**Supplemental Figure 1-7**

**
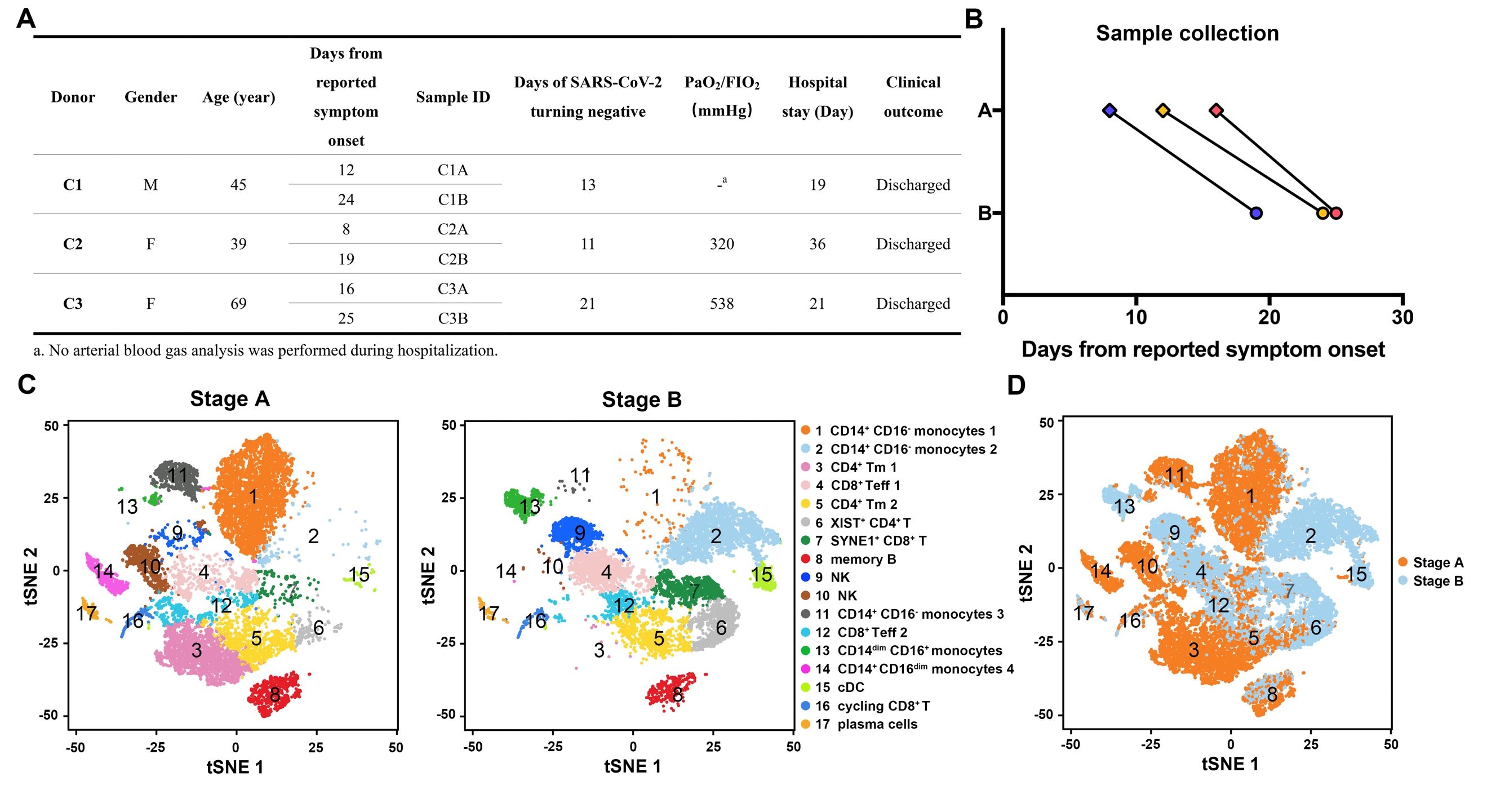
**

**Figure S1. Demographic and clinical information of COVID-19 patients**

**(A)** Demographic and clinical information of COVID-19 patients used in the current study. **(B)** Time points of sample collection in the three patients. Samples of patient C1 were represented as yellow square (stage A) and yellow spot (stage B), samples of patient C2 were represented as blue square (stage A) and blue spot (stage B), samples of patient C3 were represented as red square (stage A) and red spot (stage B). **(C)** tSNE plots of immune cells in stage A and stage B respectively. 17 clusters distributed in the two stages as indicated. **(D)** Distribution of clusters in stage A and stage B. Cells in stage A are indicated as orange, and cells in stage B are indicated as light blue.


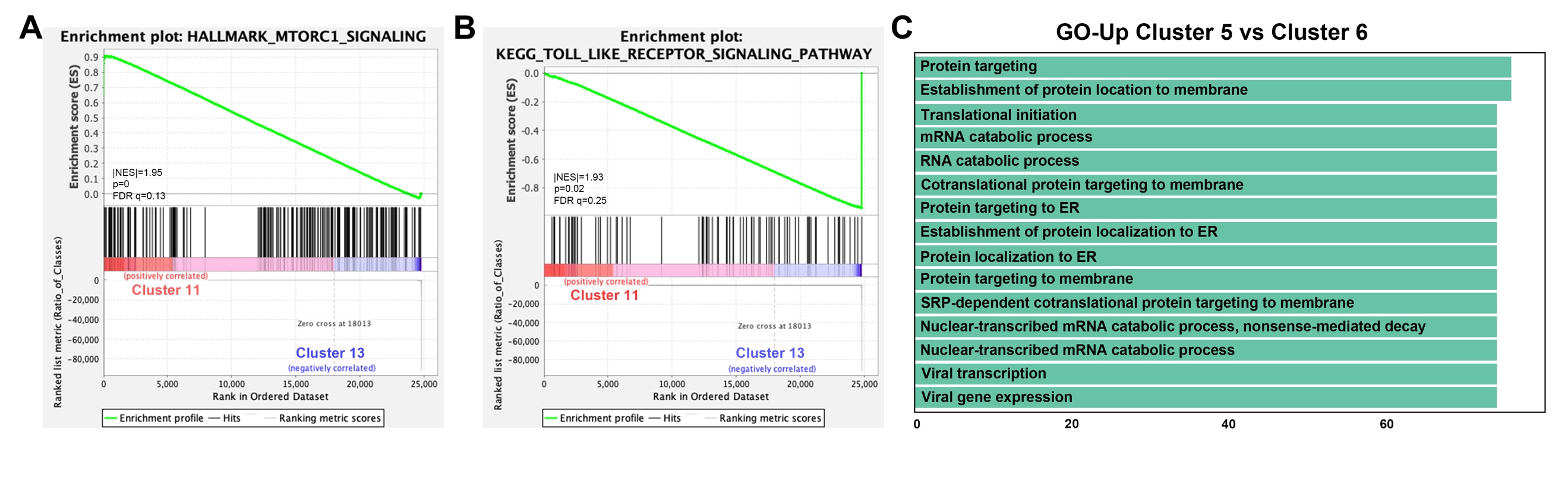


**Figure S2. GSEA analysis of CD14^+^ CD16^-^ monocytes 3 (Cluster 11) and CD14^dim^ CD16^+^ monocytes (Cluster 13).**

**(A)** Gene set enriched in CD14^+^ CD16^-^ monocytes 3 (Cluster 11)-mTORC1 signaling. |NES| = 1.95, p = 0, FDR q = 0.13. **(B)** Gene set enriched in CD14^dim^ CD16^+^ monocytes (Cluster 13)-toll like receptor pathway. |NES| = 1.93, p = 0.02, FDR q = 0.25. **(C)** Go analysis with genes highly expressed in memory CD4^+^ T cells 2 (Cluster 5) comparing with Cluster 6- XIST^+^ CD4^+^ T cells (Cluster 6).


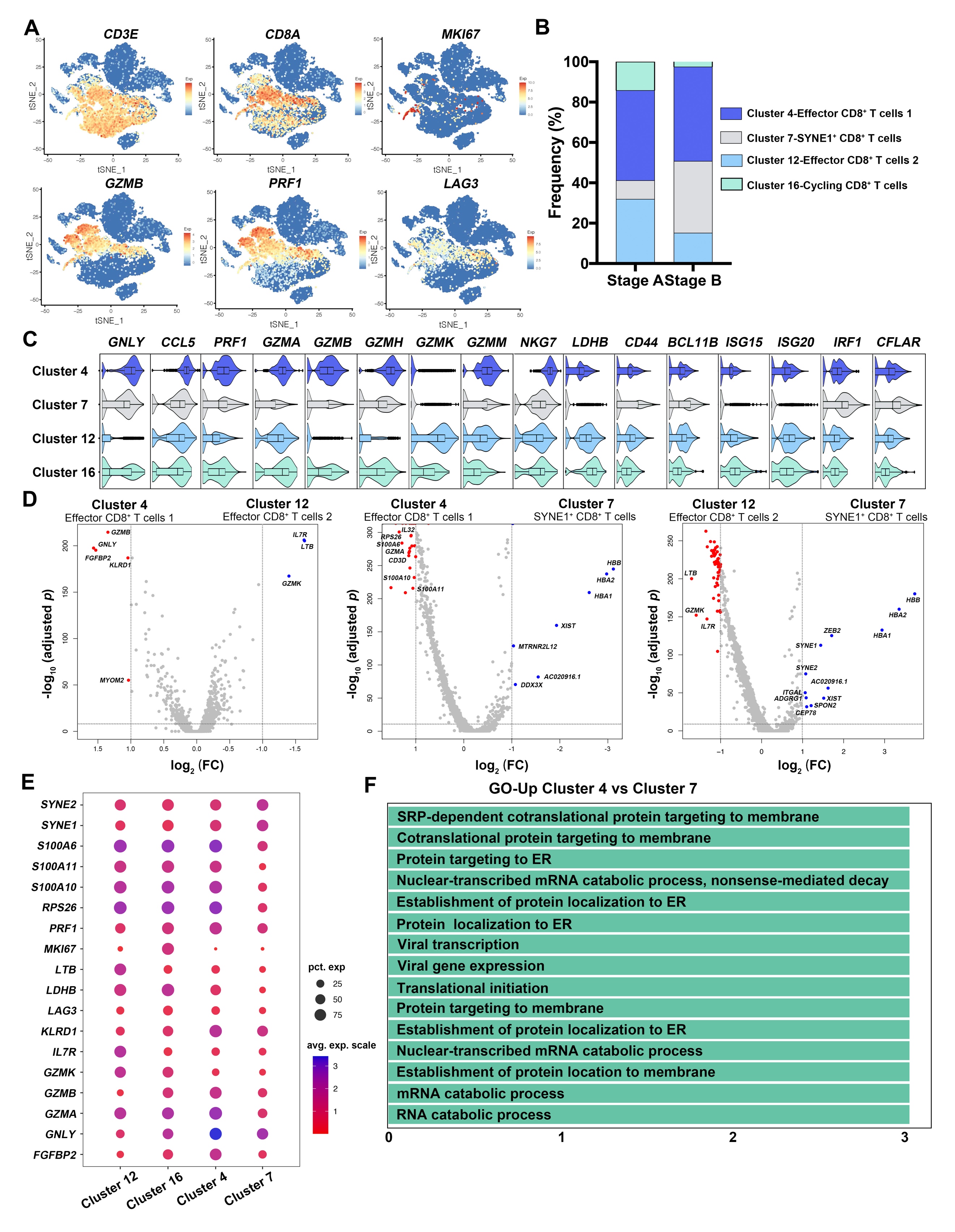


**Figure S3. Features of CD8^+^T cells in different stages in COVID-19 patients**

**(A)** Feature plots of characteristic genes of CD8^+^T cells were represented via tSNE. **(B)** Frequency distribution of the four CD8^+^T cell clusters, Cluster 4 (effector CD8^+^T cells 1), Cluster 7 (SYNE1^+^CD8^+^T cells), Cluster 12 (effector CD8^+^T cells 2), Cluster 16 (cycling CD8^+^T cells), in each stage. **(C)** Genotypes of the four CD8^+^T cell clusters. **(D)** Volcano plots of Cluster 4 (effector CD8^+^T cells 1) versus Cluster 12 (effector CD8^+^T cells 2), Cluster 4 (effector CD8^+^T cells 1) versus Cluster 7 (SYNE1^+^CD8^+^T cells), Cluster 12 (effector CD8^+^T cells 2) versus Cluster 7 (SYNE1^+^CD8^+^T cells). Differential expressed gene were defined with threshold of fold change ≥ 2 and p value < 0.05 and represented as red or blue. **(E)** Signature genes generated from differential gene expression analysis. Size of dots were related with percentage of gene expression. Bigger and bluer dots represented higher average gene expression. **(F)** Go analysis with genes highly expressed in Cluster 4 comparing with Cluster 7.


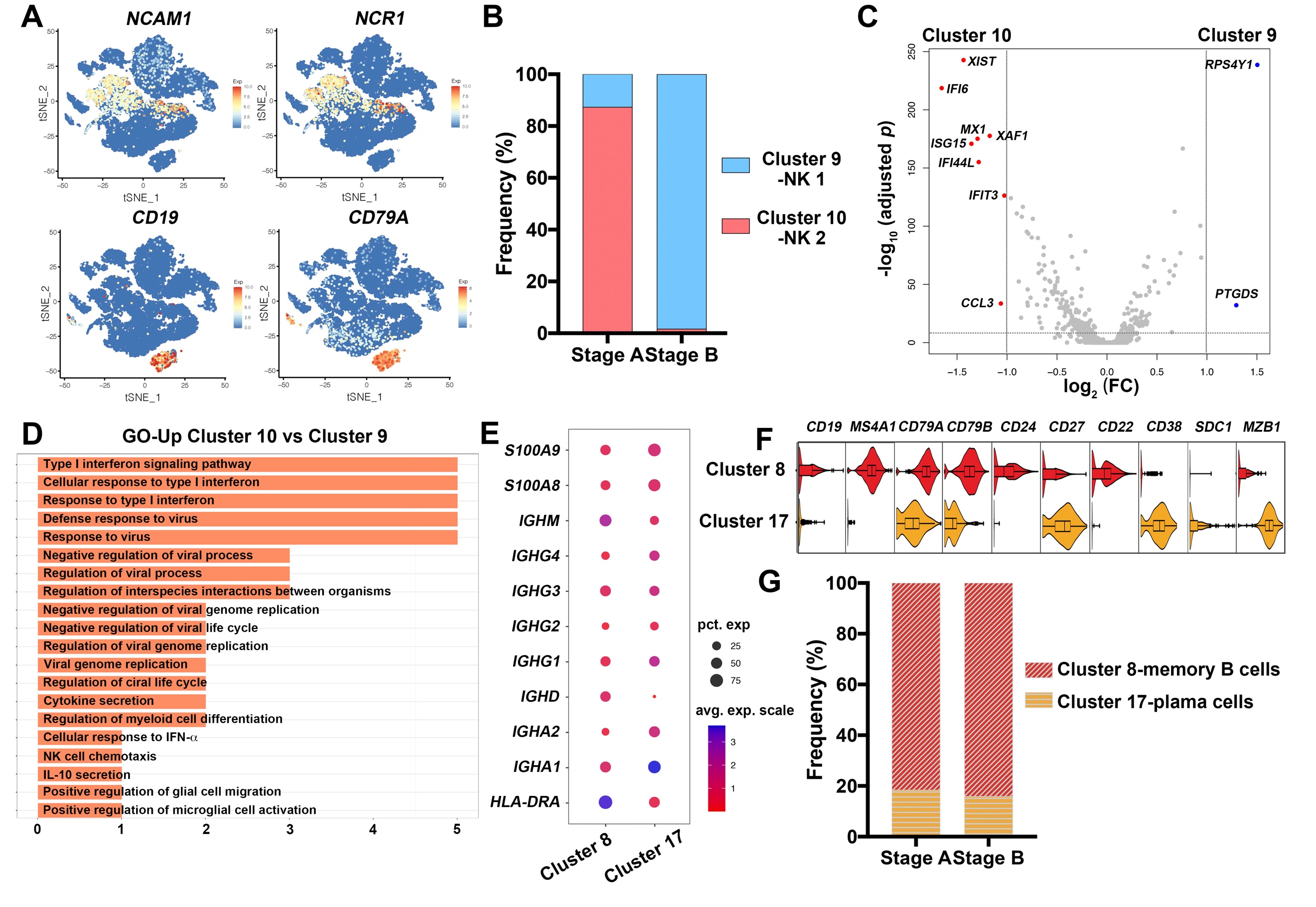


**Figure S4. Immune phenotypes changed in NK cells but not B cells**

**(A)** Feature plots of characteristic genes of NK and B cells were represented via tSNE. **(B)** Frequency distribution of the two NK cell clusters, Cluster 9 and Cluster 10, in each stage. **(C)** Volcano plots of Cluster 10 (NK cells 2) versus Cluster 9 (NK cells 1). Differential expressed gene were defined with threshold of fold change ≥ 2 and p value < 0.05 and represented as red or blue. **(D)** Go analysis with genes highly expressed in Cluster 10 comparing with Cluster 9. **(E)** Signature genes generated from differential gene expression analysis. Size of dots were related with percentage of gene expression. Bigger and bluer dots represented higher average gene expression. **(F)** Genotypes of the two B cell clusters. **(G)** Frequency distribution of Cluster 8 (memory B cells) and Cluster 17 (plasma cells) in each stage.


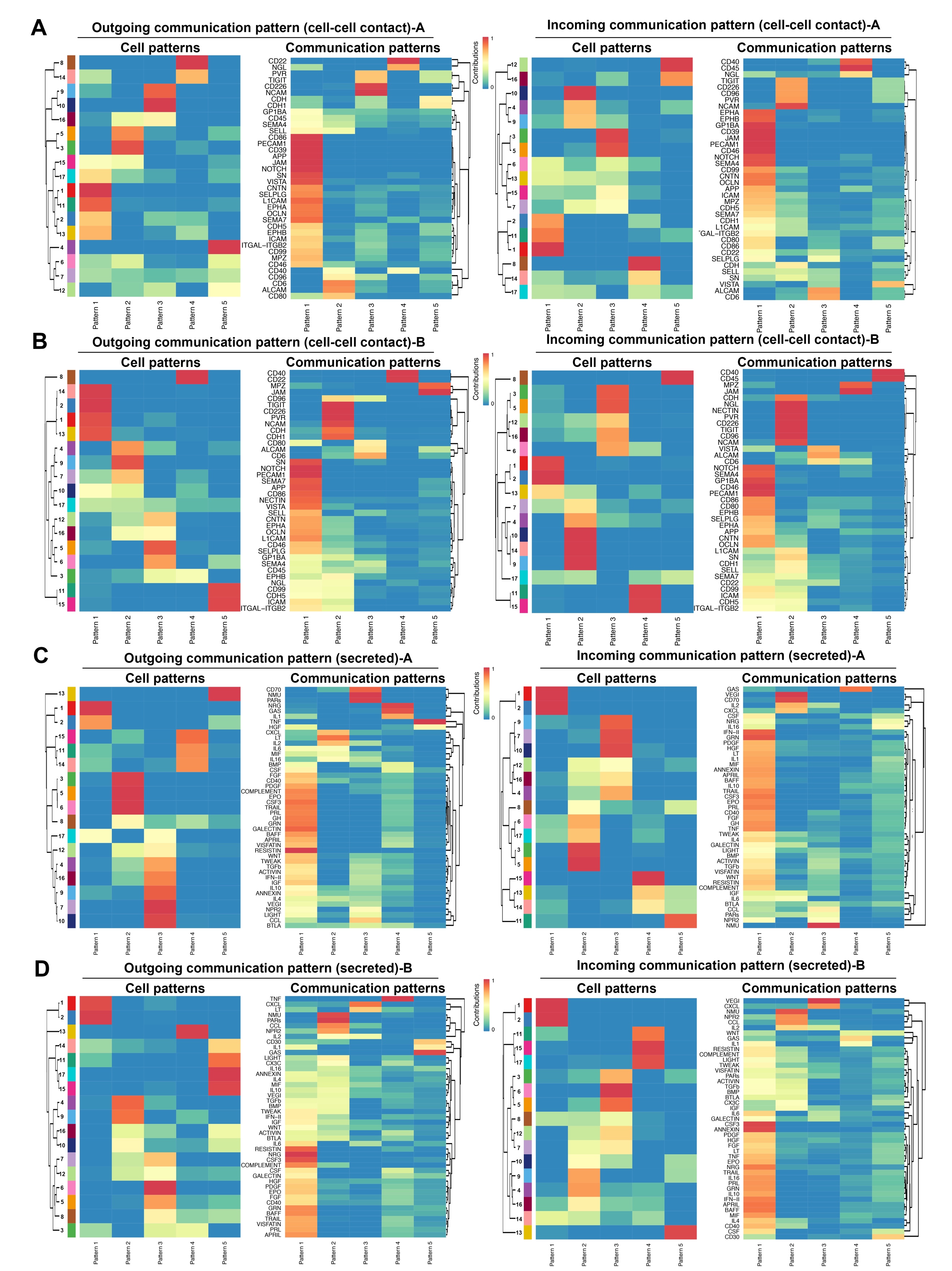


**Figure S5. Cellular communications in immune cells**

**(A)** Outgoing and incoming communication patterns of cell-cell contact in immune cells in stage A. **(B)** Outgoing and incoming communication patterns of cell-cell contact in immune cells in stage B. **(C)** Outgoing and incoming communication patterns of secreted signaling in immune cells in stage A. **(D)** Outgoing and incoming communication patterns of secreted signaling in immune cells in stage B.


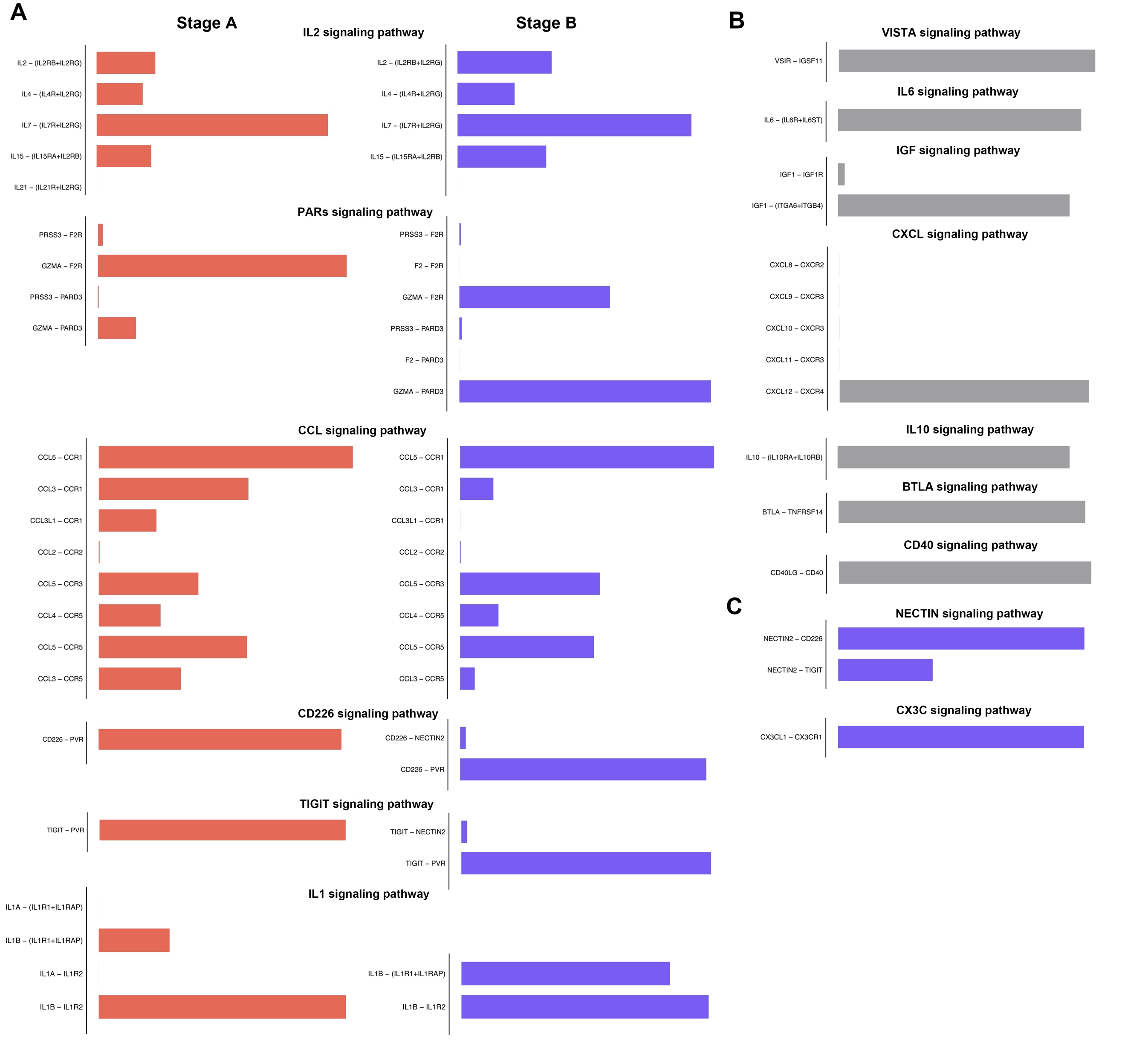


**Figure S6. Contribution of each ligand and receptor in individual signaling pathway.**

**(A)** Signaling pathways that were differed in distribution and contribution of ligand and receptor in stage A and stage B. **(B)** Signaling pathways that were similar in distribution and contribution of ligand and receptor in both stage A and stage B. **(C)** Signaling pathways that were unique in distribution and contribution of ligand and receptor in stage B.


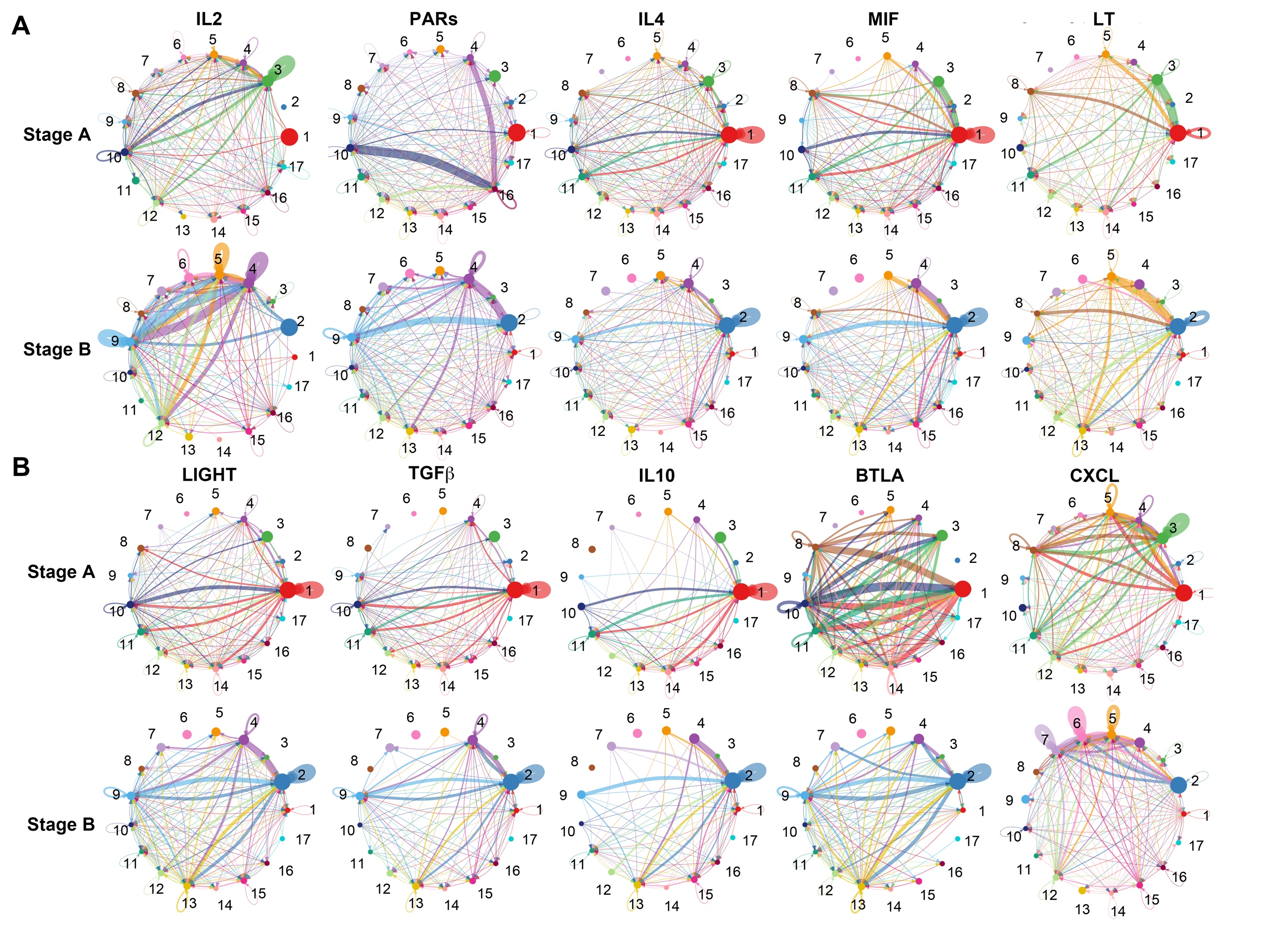


**Figure S7. Communication network of regulatory and chemokine signals in stage A and stage B.**

**(A)** Communication network of activated signals in stage A and stage B. **(B)** Communication network of regulatory and chemokine signals in stage A and stage B. Signals sent by specific cluster were drew with the same color as indicated. Target of signaling pathways were pointed by line arrows. Thickness of lines indicated the degree of the signal.
